# Use of a nanosecond Q-switched 1064 nm Nd: YAG laser for photoaging management in Asian skin: a retrospective study

**DOI:** 10.1007/s10103-026-04973-7

**Published:** 2026-08-10

**Authors:** Elena Zappia, Mario Sannino, Fortunato Cassalia, Umberto Santaniello, Eugenio Provenzano, Massimo Vitale, Marco Marcasciano, Simone Ribero, Annunziata Dattola, Steven Paul Nisticò, Giovanni Cannarozzo

**Affiliations:** 1 Department of Medical and Cardiovascular Sciences, Sapienza University of Rome, Rome, Italy; 2 Department of Health Sciences, Magna Graecia University, Catanzaro, Italy; 3 Dermatology Unit, Department of Medicine, University of Padua, Padova, Italy; 4 Department of Medical Sciences, University of Turin, Turin, Italy; 5 Dermatology Unit, Azienda Ospedaliera di Cosenza, Cosenza, Italy; 6 Private Practice, Bologna, Italy; 7 Plastic and Reconstructive Surgery Unit, Department of Experimental and Clinical Medicine, Magna Graecia University, Catanzaro, Italy

**Keywords:** Laser, Photoaging, Asian skin, Wrinkles, Aesthetic dermatology

## Abstract

Facial wrinkles are a major concern in aesthetic dermatology, particularly in patients with darker phototypes, in whom laser treatments require careful parameter selection to minimize pigmentary adverse events. This retrospective study evaluated the effectiveness and tolerability of a nanosecond Q-switched 1064 nm Nd laser for the management of facial photoaging in Asian patients. Fifty Asian patients with Fitzpatrick skin types III–IV and facial wrinkles underwent three monthly treatment sessions with a Q-switched 1064 nm Nd: YAG laser. Clinical outcomes were assessed using the Fitzpatrick Wrinkle and Elastosis Scale, standardized pre- and post-treatment photographs, pain Visual Analogue Scale, and adverse event monitoring. Mean Fitzpatrick Wrinkle and Elastosis Scale score significantly decreased from 4.54 ± 1.55 at baseline to 3.42 ± 0.97 at three-month follow-up after the last treatment, corresponding to a mean absolute reduction of 1.12 ± 0.75 points. Forty-one patients showed an improvement of at least one FWS point, while thirteen patients improved by two or more points. Nine patients remained stable, and no clinical worsening was observed. Treatment was well tolerated, with a mean pain score of 2.0 ± 0.78. Mild transient erythema occurred in four patients, while no cases of blistering, scarring, burns, hypopigmentation, or post-inflammatory hyperpigmentation were recorded. Nanosecond Q-switched 1064 nm Nd laser treatment may represent a well-tolerated option for mild-to-moderate facial photoaging in Asian skin. However, the retrospective design, lack of a control group, and absence of instrumental objective measurements should be considered when interpreting these findings.

## Introduction

Cutaneous aging begins after age 25 and is due to a series of structural changes; it is a complex and multifactorial process that involves both intrinsic factors (genetics, hormone, and metabolic) and extrinsic factors (long-term exposure to solar ultraviolet (UV), air pollution, smoking, poor nutrition, and chemicals) [1] that generate thinning and sagging of the dermis and epidermis.

Over the years, hypoestrogenism, the reactive oxygen species (ROS), and exposure to UV radiation caused structural and functional changes in all layers of the skin due to reduced expression of collagen, elastic fibers, and glycosaminoglycans. Degradation of collagen and abnormal elastin accumulation in the superficial dermis may cause loss of thickness and elasticity with the formation of wrinkles. At the same time, the sebaceous glands also decrease their activity; this causes a further weakening of the skin, which is thus more sensitive and less protected [2].

The consequences of these changes are visible: the skin begins to be less compact, loses volume, and manifests wrinkles.

Skin photoaging signs treatment involves the use of different cosmetic procedures and technologies.

The study aimed to evaluate collagen remodeling and clinical improvement in Asian skin using a fractional nanosecond Q-switched 1064 nm Nd: YAG laser, with the goal of minimizing downtime and pigmentary adverse events. Some essential characteristics must be considered before skin laser treatment on Asians. Aging skin in Asians tends to occur at a later age than that in Caucasians [3]. Asian population includes skin phototypes ranging from Fitzpatrick types III to V and is often more pigmented than Caucasian skin, resulting in interference by epidermal melanin when using lasers to treat dermal lesions: adverse pigmentary reactions, especially post-inflammatory hyperpigmentation, and keloids are more frequent [4, 5].

## Materials and methods

The study included 50 Asian patients (mean age 52.52 ± 10.62 years), with Fitzpatrick skin types III-IV and facial wrinkles. These patients were treated in the Dermatology Department of the University of Rome “Tor Vergata” and the Magna Graecia University Dermatological Unit in Catanzaro, Italy. Patients were treated with the Q-Switched 1064 nm Nd: YAG laser (Smart PICO^®^; DEKA M.E.L.A. S.p.A., Calenzano, Italy). Informed consent on the procedure’s risks was obtained from all patients.

A transparent conductive gel was used in all procedures. Exclusion criteria for the study were the following: patients hypersensitive to light in the near-infrared wavelength region; the use of medication that is known to increase sensitivity to sunlight; the use of anticoagulant and/or immunosuppressant; patients with seizure disorders triggered by light; pregnant patients; patients with personal or family history of skin cancer; patients who have been exposed to the sun in three weeks prior to treatment (for any skin type); the presence of tattoos or skin disorders on the areas to be treated. Sessions were performed at a 30-day interval.

Laser parameters were selected according to Fitzpatrick phototype and treatment endpoint. Treatments were performed using a 1064 nm Q-switched Nd: YAG laser in nanosecond mode, with a pulse duration of 6 ns, an 8 mm fractional handpiece, a repetition rate of 1–10 Hz, and fluence ranging from 0.7 to 1.3 J/cm² for phototype III patients and 0.5–1.0 J/cm² for phototype IV patients.

Treatment was carried out by passing the handpiece in contact with the skin surface, without excessive pressure, with consecutive spots and no overlapping on affected areas. Two laser passes were performed. Areas close to the bone surface (forehead, cheekbone, etc.) were treated with only one passage to avoid minor burns and hyperpigmentation. After treatment, the skin was cooled with cold water-soaked gauze, and a non-steroidal anti-inflammatory cream was applied. Applying topical anesthetics was optional: it was used in only three patients and wholly removed before treatment. Post-operative recommendations included total block mineral sunscreens for the whole treatment and follow-up period.

The efficacy and safety of the study were assessed using three methods:

i. Comparison of digital photographs before and three months after the last treatment.
ii. Fitzpatrick Wrinkle and Elastosis Scale (FWS) assessed by the investigator as compared to baseline.
iii. Visual Analogue Scale (VAS) of 5 points (0 – None, 1 – Slight pain, 2 – Moderate pain, 3 – Severe pain, 4 – Intolerable pain) to evaluate safety and tolerance.

The appearance of side effects such as blistering, scarring, burns, hypopigmentation, or hyperpigmentation have also been monitored. Statistical analysis was performed using paired Student T-test. This study was approved by the ethical committee Calabria Centro with reference number 2019/373.

## Results

A total of 50 Asian patients were included in the analysis. The mean age was 52.52 ± 10.62 years, with an age range of 31–72 years. Forty-five patients were female and five were male. Fitzpatrick phototype III was observed in 29 patients, while phototype IV was observed in 21 patients. Baseline demographic and clinical characteristics, including FWS scores before and after treatment, pain VAS, and adverse events, are summarized in Table 1.

**Table 1:** Patients characteristics

ID	Sex	Age	Photo type	FWS before	FWS after treatment	Pain VAS	Side Effects
				treatment			
1	F	50	3	4	3	3	None
2	F	43	3	4	2	2	None
3	F	37	4	3	3	2	None
4	F	61	3	6	4	1	None
5	F	48	4	4	3	2	None
6	M	52	3	4	3	3	Erythema
7	F	38	4	2	2	1	None
8	F	47	4	5	4	2	None
9	F	53	3	4	3	3	None
10	F	48	4	3	2	2	None
11	M	43	4	3	3	1	None
12	F	58	4	4	3	2	None
13	F	71	3	6	4	2	None
14	F	63	4	4	3	3	Erythema
15	F	64	3	6	5	2	None
16	F	57	3	5	4	3	None
17	F	34	3	2	2	3	None
18	F	47	3	4	3	1	Erythema
19	F	64	4	7	5	3	None
20	F	53	3	5	4	2	None
21	F	58	4	5	4	3	None
22	M	53	4	4	3	3	None
23	F	49	3	3	2	2	None
24	F	61	4	5	4	3	None
25	F	64	3	6	4	2	None
26	F	72	4	8	5	2	None
27	F	63	3	6	4	1	None
28	F	54	3	4	3	3	None
29	F	58	3	5	3	3	None
30	F	53	4	4	3	3	None
31	F	64	4	6	4	2	Erythema
32	F	48	4	3	3	1	None
33	F	42	3	3	2	1	None
34	F	38	3	4	3	1	None
35	F	67	3	8	5	2	None
36	F	58	4	5	4	2	None
37	F	45	3	3	3	3	None
38	F	56	4	4	3	2	None
39	M	34	3	2	2	1	None
40	F	39	3	4	3	2	None
41	F	65	3	7	5	1	None
42	F	54	4	5	4	1	None
43	F	67	4	8	6	2	None
44	F	45	3	5	4	1	None
45	F	49	4	4	3	2	None
46	F	59	3	5	4	3	None
47	F	69	4	7	5	2	None
48	M	39	3	4	3	1	None
49	F	41	3	3	3	1	None
50	F	31	3	2	2	1	None

A statistically significant improvement in FWS score was observed after treatment. Mean FWS score decreased from 4.54 ± 1.55 at baseline to 3.42 ± 0.97 at the three-month follow-up after the last treatment session, with a mean absolute reduction of 1.12 ± 0.75 points. This reduction was statistically significant using paired Student’s t-test (*p* ≤ 0.001).

**Fig. 1:**
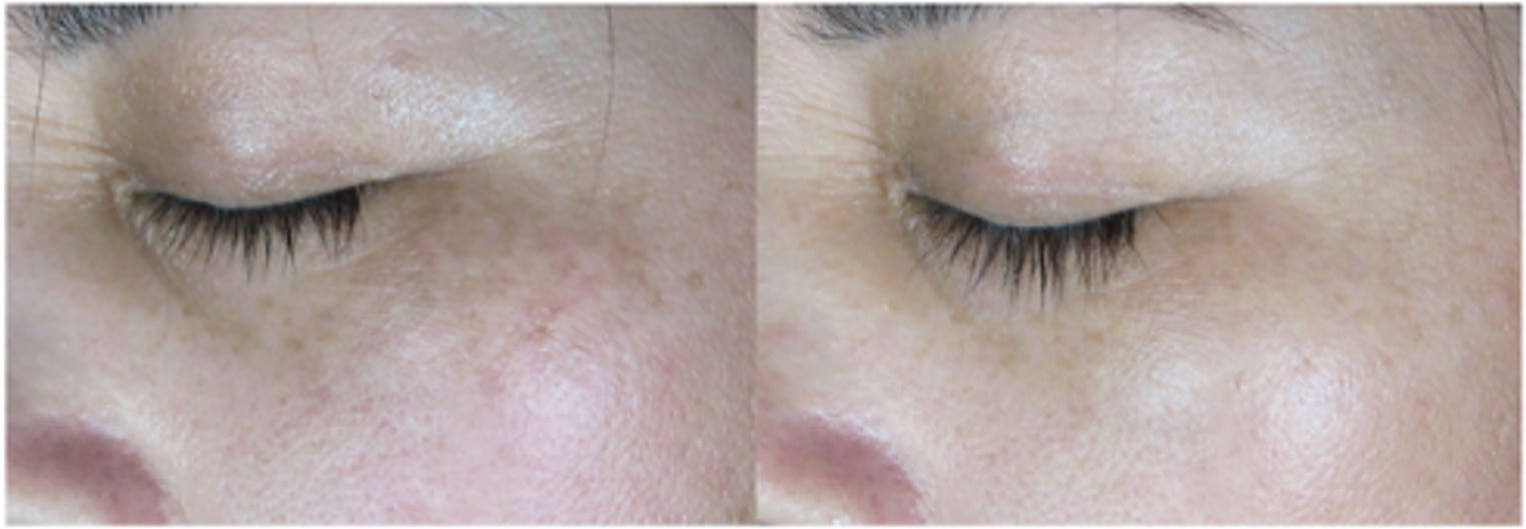
Patient before (left) and after (right) all treatments

**Fig. 2:**
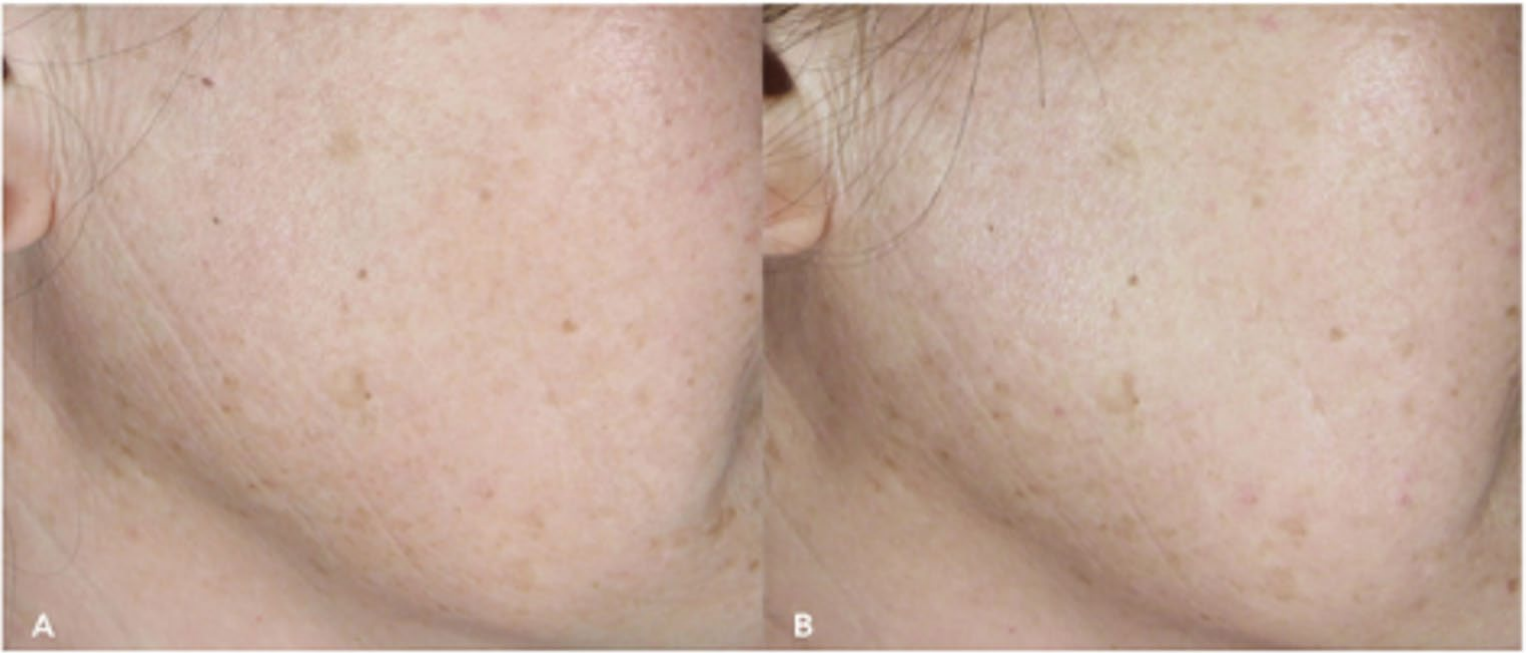
Patient before (**A**) and after (**B**) all treatments

**Fig. 3:**
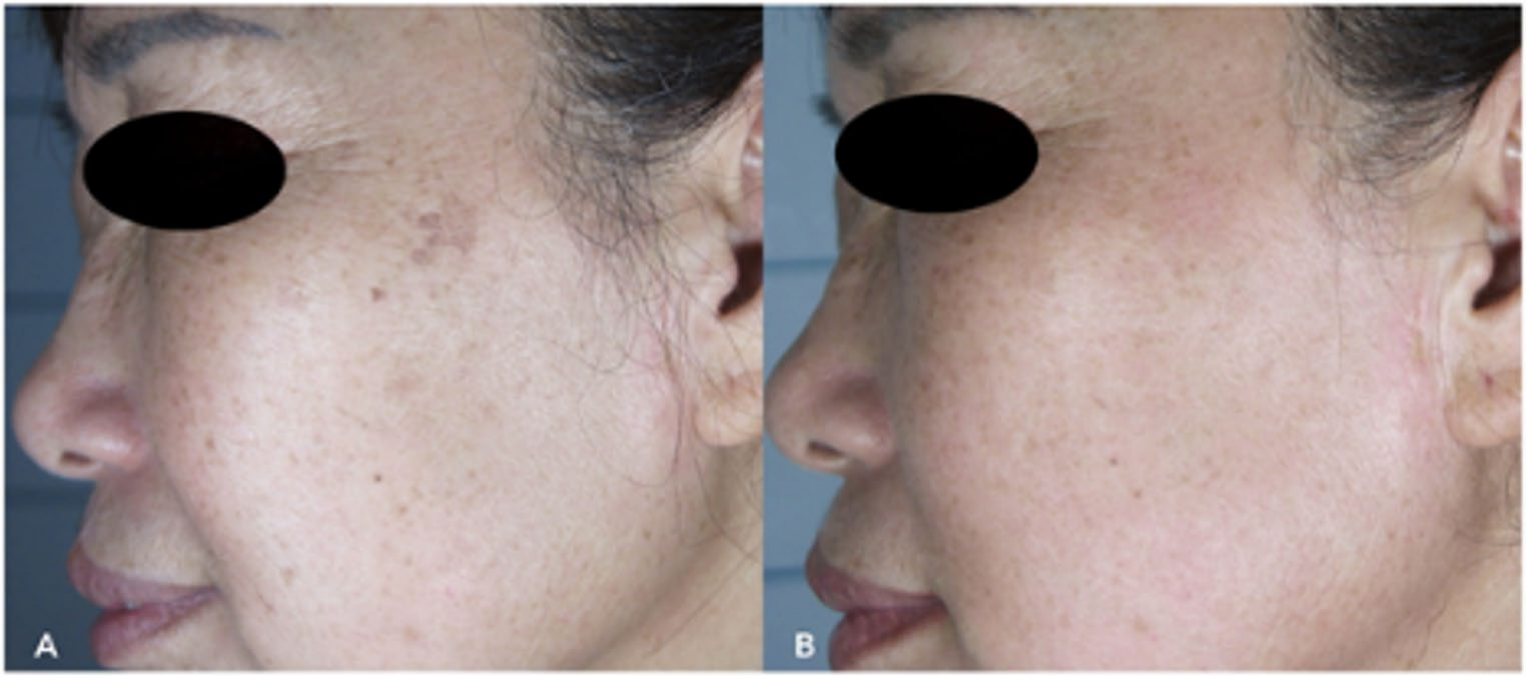
Patient before (**A**) and after (**B**) all treatments

Overall, 41 of 50 patients showed an improvement of at least one FWS point, corresponding to 82% of the study population. Thirteen patients improved by two or more FWS points, while nine patients remained stable. No patient showed worsening of FWS score after treatment. Representative pre- and post-treatment clinical photographs are shown in Figs. 1, 2 and 3.

Treatment was generally well tolerated. The mean pain VAS score was 2.0 ± 0.78, indicating mild-to-moderate discomfort. Mild transient erythema occurred in four patients and resolved without sequelae. No cases of blistering, scarring, burns, hypopigmentation, post-inflammatory hyperpigmentation, or other persistent adverse events were observed during the follow-up period (Table 1).

## Discussion

The findings of this study are clinically relevant because the treatment of facial photoaging in Asian skin requires a careful balance between efficacy and safety. Asian patients commonly present with Fitzpatrick phototypes III–IV and are more prone to pigmentary adverse events after laser procedures. Therefore, laser modalities that can improve skin texture and wrinkles while minimizing epidermal disruption and downtime are particularly valuable in this population.

Treatment of facial wrinkles has become a significant issue in cosmetic dermatology. Superficial wrinkles are associated with textural changes in the skin surface caused by intrinsic and extrinsic aging. Skin changes associated with chronological aging or photoaging, such as wrinkles, laxity, and changes in pigmentation, often prompt patients to seek cosmetic procedures to improve the appearance of their skin. Facial wrinkles can be classified into two types: static and dynamic. Static wrinkles are always visible even when all facial muscles are at rest, as they develop due to premature or natural aging processes. Dynamic wrinkles, on the other hand, appear temporarily after facial expressions [6].

The intrinsic aging of the skin begins after age 25 and is due to a series of structural changes that generate thinning and sagging of the dermis and epidermis [2]. The degradation of collagen and the abnormal accumulation of elastin that occur over the years in the superficial dermis can cause loss of thickness and elasticity, leading to the formation of wrinkles [7–9]. Wrinkles can also be classified into gravitational wrinkles, expression lines, actinic wrinkles, and sleep wrinkles. Gravitational wrinkles are caused by the action of gravity on the skin, especially when it becomes less elastic and less resistant to traction because of aging-related changes [10]. Expression lines underline facial expressions and are more evident in individuals with pronounced facial mimic activity. Actinic wrinkles are related to the cumulative effect of ultraviolet radiation and are typically visible in chronically sun-exposed areas, such as the face and neck. Finally, sleep wrinkles are caused by recurrent mechanical compression during sleep.

Wrinkles become more visible after the age of 40, when the production of hyaluronic acid, which contributes to skin hydration and elasticity, also decreases [11]. The reduction in estrogen levels associated with menopause further contributes to lower collagen production, resulting in loss of facial volume, firmness, and elasticity [12]. These mechanisms explain why photoaging is a multifactorial process and why treatments aimed at inducing dermal remodeling may provide clinical benefit.

Several topical and physical treatments are currently available for facial rejuvenation. Topical treatments include cosmetic and cosmeceutical products intended to improve hydration, stimulate cell renewal, or counteract oxidative stress, such as formulations containing water, lipids, hyaluronic acid, anti-aging compounds, and nutrients [13]. However, topical therapies usually provide gradual and limited improvement, especially in patients with established wrinkles or dermal remodeling.

Among physical treatments, ablative and non-ablative lasers are widely used for skin rejuvenation. Ablative lasers vaporize tissue and are therefore more aggressive than non-ablative lasers, which leave the epidermal surface largely intact [14]. After ablative laser treatment, patients may report erythema, burning sensation, prolonged downtime, and a higher risk of pigmentary alterations. Conversely, the potential risks associated with non-ablative lasers are significantly lower than those associated with ablative procedures [15]. The main advantage of non-ablative devices is the marked reduction in downtime compared with CO2 and erbium lasers [16, 17]. These devices may also be more suitable for patients with darker skin phototypes because, unlike ablative lasers, they are less likely to induce post-inflammatory hyperpigmentation, as the less extensive thermal injury causes reduced melanocytic stimulation [18]. The incidence of transient post-inflammatory hyperpigmentation following CO2 laser resurfacing ranges from 3% to 7% in the overall population and may reach 68% among patients with skin type IV and higher [19]. Non-ablative lasers therefore offer targeted treatments focused on textural improvement, acne-related changes, and overall skin aging, with a more favorable safety profile [17].

Before performing laser treatment, it is also necessary to consider the morphological and structural differences between Asian and Caucasian skin [20]. Asian skin is generally more pigmented than Caucasian skin, usually corresponding to Fitzpatrick phototypes III–IV. This is not due to a higher number of melanocytes, but rather to larger and more active melanosomes [21]. As a result, side effects including post-inflammatory hyperpigmentation and hypopigmentation are more likely after laser treatment, because of greater absorption of laser energy and possible melanosome damage [22]. These pigmentary changes may also occur several months after treatment [23, 24]. In addition, Asian skin may have a greater tendency toward hypertrophic scars and keloids after cutaneous trauma [25]. Although Asian patients often show greater baseline pigmentation, photoaging tends to occur later than in Caucasian patients and is generally characterized by reduced wrinkle formation and prominent pigmentary changes [26, 27].

In this context, the safety profile observed in the present study is particularly important. Only four patients developed mild transient erythema, and no cases of post-inflammatory hyperpigmentation, hypopigmentation, scarring, burns, or blistering were reported. This suggests that, when conservative parameters are selected according to phototype and treatment endpoint, fractional Q-switched 1064 nm Nd laser treatment may be a suitable approach for facial rejuvenation in Asian patients. The absence of pigmentary complications is particularly relevant considering the increased susceptibility of darker phototypes to post-laser dyschromia.

Several studies in the literature have evaluated the role of 1064 nm Q-switched Nd laser systems in skin rejuvenation and dermal remodeling. Goldberg was the first to use the 1064 nm Q-switched Nd laser for facial rejuvenation in 1997, demonstrating complete re-epithelialization 3–6 days earlier than in sites treated with CO2 laser resurfacing, without pigmentary changes [28]. Lee et al. later demonstrated significant improvement in skin texture, skin tone, and sebum secretion after 1064 nm Q-switched Nd treatment in Asian patients [29]. These findings are consistent with the present study, in which clinical improvement was observed in FWS scores and photographic evaluation, with minimal downtime and a favorable tolerability profile.

The 1064 nm Q-switched Nd laser may promote dermal remodeling through photoacoustic and controlled photothermal effects. Previous studies have suggested that this wavelength can stimulate heat shock protein 70 and procollagen 1 expression in dermal dendritic cells, potentially contributing to collagen deposition in the papillary and upper reticular dermis [30, 31]. Microscopic thermal injuries may initiate a wound-healing response and subsequent neocollagenesis through activation of the TGFβ1/SMAD3/p38MAPK pathway [32]. Yang et al. also demonstrated that 1064 nm Q-switched Nd laser treatment may regulate skin barrier function and collagen synthesis through microRNA-mediated modulation of the TGFβ1/SMAD3/p38MAPK pathway [33, 34]. Furthermore, 1064 nm Q-switched Nd laser exposure has been associated with changes in keratinocyte-fibroblast interaction and increased expression of molecular rejuvenation markers, supporting its potential role in epidermal differentiation and dermal remodeling [35, 36].

The present findings are also in line with the broader use of Q-switched and picosecond-domain laser technologies for pigmentary and rejuvenation indications. These technologies allow energy delivery with limited thermal diffusion, thereby reducing damage to surrounding tissues and lowering the risk of adverse effects. Different spot sizes and fractional handpieces provide treatment flexibility according to the target, including wrinkles, scars, pigmentary lesions, and tattoos [37, 38]. In the present study, the use of a fractional 1064 nm Q-switched approach allowed treatment of facial photoaging in patients with darker phototypes while maintaining a conservative safety profile.

Another strength of this study is the homogeneous treatment protocol and follow-up schedule. All patients underwent three monthly sessions and were evaluated three months after the last treatment. The use of FWS allowed a standardized clinical assessment of wrinkle severity, while pain VAS and adverse event monitoring provided additional information regarding tolerability and safety. Nevertheless, despite the statistically significant improvement observed, the clinical magnitude of the effect should be interpreted cautiously. A mean FWS reduction of 1.12 points indicates a measurable improvement, but not a complete correction of photoaging. Therefore, this treatment should be considered a progressive, low-downtime rejuvenation procedure rather than an alternative to more aggressive resurfacing techniques in patients with severe wrinkles or marked skin laxity.

This study has several limitations. First, its retrospective design limits the strength of the conclusions. Second, the sample size was relatively small and no control group was included. Third, outcome assessment was based mainly on FWS and standardized clinical photographs. Although these tools are clinically useful, they are less objective than instrumental measurements. The main limitation of this study is the lack of instrumental objective evaluation, such as three-dimensional skin profilometry, cutometry, high-frequency ultrasound, optical coherence tomography, or standardized skin analysis systems. Therefore, although the FWS reduction and photographic improvement suggest clinical benefit, the results should be interpreted as preliminary. Finally, the follow-up period was limited to three months after the last treatment session, so longer follow-up is needed to assess the durability of the clinical improvement and the possible delayed occurrence of pigmentary adverse events.

## Conclusions

In conclusion, fractional Q-switched 1064 nm Nd laser treatment was associated with a statistically significant improvement in FWS scores and a favorable safety profile in Asian patients with facial photoaging. The treatment was well tolerated, with only mild transient erythema in a minority of patients and no pigmentary complications. These findings suggest that this modality may represent a useful low-downtime option for facial rejuvenation in Asian skin. However, prospective controlled studies incorporating objective instrumental assessments and longer follow-up are needed to confirm these results and better define optimal treatment protocols.

## Data Availability

No datasets were generated or analysed during the current study.

## References

[1] Khavkin J, Ellis DA (2011) Aging skin: histology, physiology, and pathology. Facial Plast Surg Clin North Am 19(2):229 – 34. 10.1016/j.fsc.2011.04.003 PMID: 21763983 10.1016/j.fsc.2011.04.003 21763983

[2] Zouboulis CC, Coenye T, He L, Kabashima K, Kobayashi T, Niemann C, Nomura T, Oláh A, Picardo M, Quist SR, Sasano H, Schneider MR, Törőcsik D, Wong SY (2022) Sebaceous immunobiology - skin homeostasis, pathophysiology, coordination of innate immunity and inflammatory response and disease associations. Front Immunol 13:1029818. 10.3389/fimmu.2022.1029818 PMID: 36439142; PMCID: PMC9686445 36439142 10.3389/fimmu.2022.1029818 PMC9686445

[3] Vashi NA, de Castro Maymone MB, Kundu RV (2016) Aging Differences in Ethnic Skin. J Clin Aesthet Dermatol 9(1):31–38 PMID: 26962390; PMCID: PMC4756870 26962390 PMC4756870

[4] Ahn HJ, Hye Suh D, Kang IH, Jun Lee S, Kyung Shin M, Yong Song K (2021) Interaction of skin with fractional picosecond laser in Asian patients. J Clin Aesthet Dermatol 14(11):14–15 PMID: 34980953; PMCID: PMC8675342 34980953 PMC8675342

[5] Nisticò SP, Sannino M, Fasano G, Marigliano M, Negosanti F, Bennardo L, Cannarozzo G (2022) Fractional Q-Switched 1064 nm Laser for Treatment of Atrophic Scars in Asian Skin. Med (Kaunas) 58(9):1190. 10.3390/medicina58091190 PMID: 36143867; PMCID: PMC9505728 10.3390/medicina58091190 PMC9505728 36143867

[6] Wong R, Geyer S, Weninger W, Guimberteau JC, Wong JK (2016) The dynamic anatomy and patterning of skin. Exp Dermatol 25(2):92–98. 10.1111/exd.12832 Epub 2015 Oct 13. PMID: 26284579 26284579 10.1111/exd.12832

[7] Shin JW, Kwon SH, Choi JY, Na JI, Huh CH, Choi HR, Park KC (2019) Molecular Mechanisms of Dermal Aging and Antiaging Approaches. Int J Mol Sci 20(9):2126. 10.3390/ijms20092126 PMID: 31036793; PMCID: PMC6540032 31036793 10.3390/ijms20092126 PMC6540032

[8] Tolone M, Bennardo L, Zappia E, Scali E, Nisticò SP (2024) New Insight into Nonablative 675-nm Laser Technology: Current Applications and Future Perspectives. Dermatol Clin 42(1):45–50. 10.1016/j.det.2023.06.004 Epub 2023 Jul 21. PMID: 37977683 37977683 10.1016/j.det.2023.06.004

[9] Avila Rodríguez MI, Rodríguez Barroso LG, Sánchez ML, Collagen (2018) A review on its sources and potential cosmetic applications. J Cosmet Dermatol 17(1):20–26. 10.1111/jocd.12450 Epub 2017 Nov 16. PMID: 29144022 29144022 10.1111/jocd.12450

[10] Piérard GE, Uhoda I, Piérard-Franchimont C (2003) From skin microrelief to wrinkles. An area ripe for investigation. J Cosmet Dermatol 2(1):21 – 8. 10.1111/j.1473-2130.2003.00012.x PMID: 17156045 10.1111/j.1473-2130.2003.00012.x 17156045

[11] Abatangelo G, Vindigni V, Avruscio G, Pandis L, Brun P (2020) Hyaluronic Acid: Redefining Its Role. Cells 9(7):1743. 10.3390/cells9071743 PMID: 32708202; PMCID: PMC7409253 32708202 10.3390/cells9071743 PMC7409253

[12] Rzepecki AK, Murase JE, Juran R, Fabi SG, McLellan BN (2019) Estrogen-deficient skin: The role of topical therapy. Int J Womens Dermatol 5(2):85–90. 10.1016/j.ijwd.2019.01.001 PMID: 30997378; PMCID: PMC6451761 30997378 10.1016/j.ijwd.2019.01.001 PMC6451761

[13] He X, Wan F, Su W, Xie W (2023) Research Progress on Skin Aging and Active Ingredients. Molecules 28(14):5556. 10.3390/molecules28145556 PMID: 37513428; PMCID: PMC10385838 37513428 10.3390/molecules28145556 PMC10385838

[14] Goldberg DJ (2003) Lasers for facial rejuvenation. Am J Clin Dermatol 4(4):225 – 34. 10.2165/00128071-200304040-00002 PMID: 12680801 10.2165/00128071-200304040-00002 12680801

[15] Bennardo L, Fasano G, Tamburi F, Zappia E, Rizzuto F, Nisticò SP, Cannarozzo G (2022) Sequential Use of CO_2_Laser Prior to Nd:YAG and Dye Laser in the Management of Non-Facial Warts: A Retrospective Study. Med (Kaunas) 58(1):115. 10.3390/medicina58010115 PMID: 35056422; PMCID: PMC8780825 10.3390/medicina58010115 PMC8780825 35056422

[16] Modena DAO, Miranda ACG, Grecco C, Liebano RE, Cordeiro RCT, Guidi RM (2020) Efficacy, safety, and guidelines of application of the fractional ablative laser erbium YAG 2940 nm and non-ablative laser erbium glass in rejuvenation, skin spots, and acne in different skin phototypes: a systematic review. Lasers Med Sci 35(9):1877–1888. 10.1007/s10103-020-03046-7 Epub 2020 May 29. PMID: 32472427 32472427 10.1007/s10103-020-03046-7

[17] Seirafianpour F, Pour Mohammad A, Moradi Y, Dehghanbanadaki H, Panahi P, Goodarzi A, Mozafarpoor S (2022) Systematic review and meta-analysis of randomized clinical trials comparing efficacy, safety, and satisfaction between ablative and non-ablative lasers in facial and hand rejuvenation/resurfacing. Lasers Med Sci 37(4):2111–2122. 10.1007/s10103-022-03516-0 Epub 2022 Feb 2. PMID: 35107665 35107665 10.1007/s10103-022-03516-0

[18] Badawi A, Tome MA, Atteya A, Sami N, Morsy IA (2011) Retrospective analysis of non-ablative scar treatment in dark skin types using the sub-millisecond Nd:YAG 1,064 nm laser. Lasers Surg Med 43(2):130-6. 10.1002/lsm.21031 PMID: 21384394 10.1002/lsm.21031 21384394

[19] Bin Dakhil A, Shadid A, Altalhab S (2023) Post-inflammatory hyperpigmentation after carbon dioxide laser: review of prevention and risk factors. Dermatol Rep 15(4):9703. 10.4081/dr.2023.9703 PMID: 38205425; PMCID: PMC10777097 10.4081/dr.2023.9703 PMC10777097 38205425

[20] Chan IL, Cohen S, da Cunha MG, Maluf LC (2019) Characteristics and management of Asian skin. Int J Dermatol 58(2):131–143. 10.1111/ijd.14153 Epub 2018 Jul 24. PMID: 30039861 30039861 10.1111/ijd.14153

[21] Iwuala C, Taylor SC (2022) Structural and functional differences in skin of colour. Clin Exp Dermatol 47(2):247–250. 10.1111/ced.14892 Epub 2021 Oct 19. PMID: 34388277 34388277 10.1111/ced.14892

[22] Lin JY, Chan HH (2006) Pigmentary disorders in Asian skin: treatment with laser and intense pulsed light sources. Skin Therapy Lett 11(8):8–11 PMID: 17024294 17024294

[23] Mar K, Khalid B, Maazi M, Ahmed R, Wang OJE, Khosravi-Hafshejani T (2024 Sep-Oct) Treatment of Post-Inflammatory Hyperpigmentation in Skin of Colour: A Systematic Review. J Cutan Med Surg 28(5):473–480 Epub 2024 Jul 29. PMID: 39075672; PMCID: PMC11514325 10.1177/12034754241265716 PMC11514325 39075672

[24] Mar K, Maazi M, Khalid B, Ahmed R, Wang OJE, Khosravi-Hafshejani T (2025 Feb) Prevention of Post-Inflammatory Hyperpigmentation in Skin of Colour: A Systematic Review. Australas J Dermatol 14. 10.1111/ajd.14432 Epub ahead of print. PMID: 39953770 10.1111/ajd.14432 PMC12062726 39953770

[25] Sadiq A, Khumalo NP, Bayat A, Genetics of Keloid Scarring (2020). Dec 8. In: Téot L, Mustoe TA, Middelkoop E, Gauglitz GG, editors. Textbook on Scar Management: State of the Art Management and Emerging Technologies [Internet]. Cham (CH): Springer; 2020. Chapter 8. PMID: 36351116

[26] Chung JH (2003) Photoaging in Asians. Photodermatol Photoimmunol Photomed 19(3):109 – 21. 10.1034/j.1600-0781.2003.00027.x PMID: 12914595 10.1034/j.1600-0781.2003.00027.x 12914595

[27] Rawlings AV (2006) Ethnic skin types: are there differences in skin structure and function? Int J Cosmet Sci 28(2):79–93. 10.1111/j.1467-2494.2006.00302.x PMID: 18492142 10.1111/j.1467-2494.2006.00302.x 18492142

[28] Goldberg DJ, Whitworth J (1997) Laser skin resurfacing with the Q-switched Nd:YAG laser. Dermatol Surg 23(10):903-6; discussion 906-7. 10.1111/j.1524-4725.1997.tb00744.x PMID: 9357499 10.1111/j.1524-4725.1997.tb00744.x 9357499

[29] Lee CN, Kim YJ, Lee HS, Kim HS (2009) Effects of Q-switched and long-pulsed 1064 nm Nd:YAG laser on enlarged facial pores. Photodermatol Photoimmunol Photomed 25(6):328 – 30. 10.1111/j.1600-0781.2009.00466.x PMID: 19906169 10.1111/j.1600-0781.2009.00466.x 19906169

[30] Dang Y, Ye X, Weng Y, Tong Z, Ren Q (2010) Effects of the 532-nm and 1,064-nm Q-switched Nd:YAG lasers on collagen turnover of cultured human skin fibroblasts: a comparative study. Lasers Med Sci 25(5):719–726. 10.1007/s10103-009-0657-4 Epub 2010 May 20. PMID: 20490593 20490593 10.1007/s10103-009-0657-4

[31] Prieto VG, Diwan AH, Shea CR, Zhang P, Sadick NS (2005) Effects of intense pulsed light and the 1,064 nm Nd:YAG laser on sun-damaged human skin: histologic and immunohistochemical analysis. Dermatol Surg. ;31(5):522-5. 10.1111/j.1524-4725.2005.31154 PMID: 15962734 10.1111/j.1524-4725.2005.31154 15962734

[32] Qin Y, Qin X, Xu P, Zhi Y, Xia W, Dang Y, Gu J, Ye X (2018) Potential role of S100A8 in skin rejuvenation with the 1064-nm Q-switched Nd:YAG laser. Lasers Med Sci 33(3):581–588. 10.1007/s10103-017-2416-2 Epub 2017 Dec 21. PMID: 29270708 29270708 10.1007/s10103-017-2416-2

[33] Yang Z, Duan X, Wang X, Li D, Xu Q, Xiang S, Guo B, He L (2022) The effect of Q-switched 1064-nm dymium-doped yttrium aluminum garnet laser on the skin barrier and collagen synthesis through miR-24-3p. Lasers Med Sci 37(1):205–214. 10.1007/s10103-020-03214-9 Epub 2021 Jan 5. PMID: 33400013; PMCID: PMC8803697 33400013 10.1007/s10103-020-03214-9 PMC8803697

[34] Yang Z, Duan X, Wang X, Xu Q, Guo B, Xiang S, Jia X, He L (2021) The effect of Q-switched 1064-nm Nd: YAG laser on skin barrier and collagen synthesis via miR-663a to regulate TGFβ1/smad3/p38MAPK pathway. Photodermatol Photoimmunol Photomed 37(5):412–421. 10.1111/phpp.12673 Epub 2021 Mar 10. PMID: 33621359 33621359 10.1111/phpp.12673

[35] Cannarozzo G, Nisticò SP, Zappia E, Del Duca E, Provenzano E, Patruno C, Negosanti F, Sannino M, Bennardo L (2021) Q-Switched 1064/532 nm Laser with Nanosecond Pulse in Tattoo Treatment: A Double-Center Retrospective Study. Life (Basel) 11(7):699. 10.3390/life11070699 PMID: 34357071; PMCID: PMC8304052 34357071 10.3390/life11070699 PMC8304052

[36] De Filippis A, Perfetto B, Guerrera LP, Oliviero G, Baroni A (2019) Q-switched 1064 nm Nd-Yag nanosecond laser effects on skin barrier function and on molecular rejuvenation markers in keratinocyte-fibroblasts interaction. Lasers Med Sci. ;34(3):595–605. 10.1007/s10103-018-2635-1 Epub 2018 Oct 1. PMID: 30276490 10.1007/s10103-018-2635-1 30276490

[37] Zappia E, Piccolo D, Del Re C, Bonan P, Guarino L, Ribero S, Galadari H, Nisticò SP (2024) Combined Efficacy of Q-Switched 785 nm Laser and Tranexamic Acid Cream in the Treatment of Melasma: A Prospective Clinical Study. Photonics 11(10):938. 10.3390/photonics11100938

[38] Brown AS, Hussain M, Goldberg DJ (2011) Treatment of melasma with low fluence, large spot size, 1064-nm Q-switched neodymium-doped yttrium aluminum garnet (Nd:YAG) laser for the treatment of melasma in Fitzpatrick skin types II-IV. J Cosmet Laser Ther. ;13(6):280-2. 10.3109/14764172.2011.630084 PMID: 21992660 10.3109/14764172.2011.630084 21992660

